# Maximum lying bout duration affects the occurrence of shoulder lesions in sows

**DOI:** 10.1186/1751-0147-51-44

**Published:** 2009-11-20

**Authors:** Elin Rolandsdotter, Rebecka Westin, Bo Algers

**Affiliations:** 1 Department of Animal Environment and Health, Swedish University of Agricultural Sciences, PO Box 234, 53223 Skara, Sweden; 2 Sigill Kvalitetssystem AB, 105 33 Stockholm, Sweden

## Abstract

Shoulder lesions are caused by tissue breakdown of the skin and/or underlying tissue as a result of long lasting pressure. The lesions are commonly seen in sows during the period of lactation and contribute to poor animal welfare as well as affecting the consumers' attitudes towards the swine industry. The aim of this study was to investigate the correlation between prolonged recumbency during early lactation and development of shoulder lesions, in particular the lying bout time. Eighteen sows of Swedish Landrace were observed for 24 hours during the day of farrowing and day 2, 4, 9 and 11 after farrowing in May 2009. The data were analysed for correlations between the duration of the longest observed uninterrupted lying bout and the prevalence of shoulder lesions recorded at weaning (week 5).

In the study, shoulder lesions were observed in eight of the eighteen sows at the time of weaning. The total lying time of the sows was highest on day 0 and day 2, when the proportion of time spent in lateral recumbency over the 24-hour period was on average 80 percent. The longest lying bout had an average duration of 6,3 hours (right side) and 7,2 hours (left side). A significant correlation (Spearman rank coefficient = 0,88; P < 0,05) was found between the duration of the longest observed uninterrupted lying bout and the occurrence of shoulder lesions on right side among well conditioned sows with a low amount of straw present at farrowing. This suggests that avoiding prolonged uninterrupted recumbency contributes to the prevention of shoulder lesions in sows.

## Background

The shoulder blade, scapula, has a prominent lateral ridge offering muscular attachment. The ridge can easily be palpated through the skin of thin animals. Shoulder lesion is a result of tissue breakdown which are caused by pressure exerted on overlying tissues by the bony ridge and external surfaces [1].

Shoulder lesions in sows range in severity from hair-loss and redness to serious muscle damage and development of granulation tissue [2]. In Denmark a common scale for classification of shoulder lesions has been established. The scale is based on a 1-4 grade system where: grade 1). The lesion is limited to the epidermis, eventually with a moderate crust. 2). The lesion involves the dermis, eventually with extensive crusting. There is little fibrosis and/or granulation tissue. 3). The lesion penetrates to the sub cutis. There is intense formation of granulation tissue. 4). Ulcer penetrating to the bone or ulcer development with periosteal bone proliferation [3].

According to several studies, the prevalence of shoulder lesions is most frequent during lactation. In one study, the prevalence of shoulder lesions was 16% in lactation and 6% in the dry period [4]. Similar results were presented in another study where the prevalence of shoulder lesions in lactating sows was remarkable higher (25%) compared with the dry sows (3,7%) [5]. The severe grades of shoulder lesions are more commonly seen during the last week of lactation [6].

Studies from human medicine show that the risk factors for developing pressure sores are multifactorial where; immobility, underlying diseases, increased body temperature, drugs, acute illness, age, nutritional status, mattress quality and moisture are contributing risk factors [7]. Also in pigs, shoulder lesions constitutes a multifactorial problem and the change in the behaviour of the sow during farrowing and early lactation with prolonged lying periods and decreased activity are risk factors of concern [8].

The combination between pressure and duration are regarded as fundamental to the development of shoulder lesions, where both the pressure from the surface as well as pressure from the body weight of the animal contributes [4,9-11]. An unrelieved pressure results in tissue breakdown of the skin and/or underlying tissue. Short periods of pressure relief can however enable tissues to recover from compression [9,10]. The precise mechanisms behind the development of shoulder lesions are yet not well known and there are several opinions about how and why pressure leads to tissue breakdown. According to one study the tissue breakdown is caused by ischemia [12]. The pressure inhibits the blood flow which results in lack of oxygen transport to the tissue. This occurs when tissue pressure exceed capillary hydrostatic pressure at 35 mm Hg. Others state that because of the obstruction of blood flow and impaired lymph drainage, it is the decreased ability for transportation of nutrients to and waste products from the cells of the tissue that causes necrosis of the skin [4,8].

According to several studies, low body condition score is a well known animal based risk factor for development of shoulder lesions [1,5,13,14]. Due to a thinner layer of fat, the scapula is more affected of pressure in thin sows, which increases the risk for developing shoulder lesions [15]. Because of a high milk production during the lactation period, the sows tend to loose weight [16]. In a Swedish study, sows with shoulder lesions of grade 3 and 4 lost more weight and more rapidly than sows with lesions of grade 1 and 2 [6]. Even though a low body condition score is referred to as a common risk factor, there are studies that show that well-conditioned sows sometimes are affected by shoulder lesions as well [14,15].

Lameness has also been proven as a risk factor. One study confirmed that sows with lameness were 16.78 times more disposed to develop shoulder lesions compared to healthy sows [16]. According to the authors, the reason for this is that the sow is spending more of the time lying down. Other risk factors that are related to long lasting recumbency are diseases like the Mastitis Metritis Agalactia complex (MMA). This is in agreement with a Swedish study where the prevalence of shoulder lesions increased due to the presence of MMA [6]. Diseases can also involve side effects such as decreased appetite and weight loss. Also age related alterations in the skeleton can lead to longer periods of recumbency [5]. Most of the sows prefer to lie on one side during the farrowing and lateral recumbency is the most common posture during early lactation [17,18]. However, there are to our knowledge no investigations concerning if individual differences in the normal lying behaviour during early lactation are correlated to the development of shoulder lesions and in particular the effects of the lying bout duration. The objective of this study was therefore to explore the effects of the duration of lying bout time on the development of shoulder lesions during early lactation.

## Materials and methods

The study was conducted in a herd with gilt production, where the breeding sows were of pure-bred Swedish Landrace. The herd was chosen because of its high prevalence of shoulder lesions. The sows were kept loose in conventional farrowing pens (2 × 3 m). 50% of the floor area consisted of a solid concrete floor and 50% of a plastic slatted floor. From the beginning, the observation group consisted of 20 sows that were expected to give birth within a time period of seven days. Since one sow got sick and another gave birth outside the trial period, the observation group in the end consisted of 18 sows. Due to another trial that was running at the same time, the observed sows were randomly assigned into two groups where one group got 15 kg straw two days before expected farrowing and the other group got a small amount of straw given each day (approximately 0.8 kg straw/day). Five days after farrowing, the remaining straw was taken out manually from the pens that were provided with large amounts of straw. Thereafter, a small amount of straw mixed with saw dust was given every day to all sows. The sows were observed with video cameras during three weeks in May 2009. The recordings were saved digitally in the software MSH-Video Server (Video Server Company).

The prevalence of shoulder lesions at the right and left shoulder was monitored when the sows entered the farrowing unit and at weaning (5 weeks after farrowing). The lesions were classified according to the Danish 4-grade scale [6]. Body condition was recorded when the sows entered the farrowing unit, where the back fat thickness was measured with ultrasonar technique (Krautkrämer, USM 22 F) at the last rib on the right side.

An ethogram of the study animals' observed behaviour is shown in table 1. The recordings were decoded in the software MSH-Video Client (Video Server Company), where the start and stop times of each behavioural bout were recorded. The first observation period included the day of farrowing (day 0), followed by observations day 2, 4, 9 and 11 after farrowing. The observations during the day of farrowing started when the first piglet was born and continued for 24 hours. Observations during the other days started at 00:00 and continued until 24:00. The recorded time values were transferred into an Excel file where different measurements for time spent in lateral recumbency were calculated, se table 2. Listed time measurements were first calculated separately for each day of observation. The longest uninterrupted lying bout observed during all days of observation (T-max) was then identified for each individual sow and each side of the body (right and left) to be included in further analysis.

**Table 1:** Ethogram

Behaviour	Definition
Lying on right side	Lying down on right side with the head and shoulder resting on the floor.
Lying on left side	Lying down on left side with the head and shoulder resting on the floor.

**Table 2:** Measurements of time spent in lateral recumbency recorded for each sow

Recorded time measure	Definition
T-day total	The proportion of time spent lying on left respectively right side during each day of observation.
T-total	The proportion of time spent lying on left respectively right side during all days of observation.
Number of lying bouts	Number of times that the sow is lying on left or right side during each day of observation.
T-day max	The duration of the longest daily uninterrupted lying bout recorded during each day of observation.
T-max	The duration of the longest uninterrupted lying bout recorded during all days of observation.

Straw supply and body condition were suspected to confound the observed effect of lying time on shoulder lesions. However, the limited number of observations did not allow for a multivariable regression model accounting for such confounding. Instead, statistical analysis was done using the CORR procedure of the statistical software SAS 9.1 (SAS Institute Inc.). Associations between T-max and the prevalence of shoulder lesions (grade 0-4) at weaning were analysed by Pearson's correlation for right and left sides separately. To control for confounding, partial correlations were also run with straw supply at farrowing (low or high) and body condition (back fat thickness) included as partial factors. Furthermore, to check for interactions, data were divided into four strata according to body condition and straw supply. Sows with a back fat thickness of > 14 mm were referred to as well conditioned whereas sows with a back fat thickness of ≤ 14 mm were referred to as thin. Associations between T-max and the prevalence of shoulder lesions was then analysed for the strata containing "thin sows with a high supply of straw" (n = 7) and "well-conditioned sows with a low supply of straw" (n = 6), using Spearman rank correlation. In the two remaining strata, "thin sows with a low supply of straw" and "well-conditioned sows with a high supply of straw", the number of animals was too low for analysis (n = 2 and 3, respectively).

## Results

### Development of shoulder lesions

**Figure 1:**
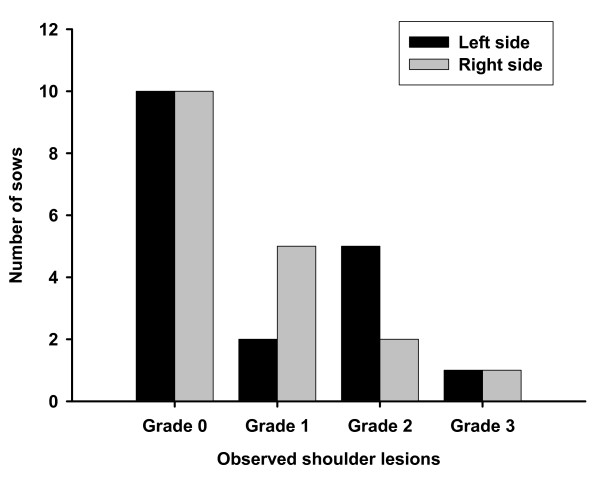
**Number of sows with shoulder lesions at the time of weaning (week 5)**.

When entering the farrowing unit, none of the sows had shoulder lesions. At weaning (week 5), shoulder lesions were observed in eight of the eighteen sows, six of the sows having lesions on both sides, see figure 1. Most common were lesions of grade 1 and 2. No lesions of grade 4 were detected.

### Measurements for number of lying bouts

The sows were most active on day 0, with the highest number of lying bouts (on average 24 bouts per sow). On day 2, activity was decreased and the lowest number of lying bouts was observed (on average 10 bouts per sow). The following days, the activity level increased again.

### Measurements for time spent in lateral recumbency

**Figure 2:**
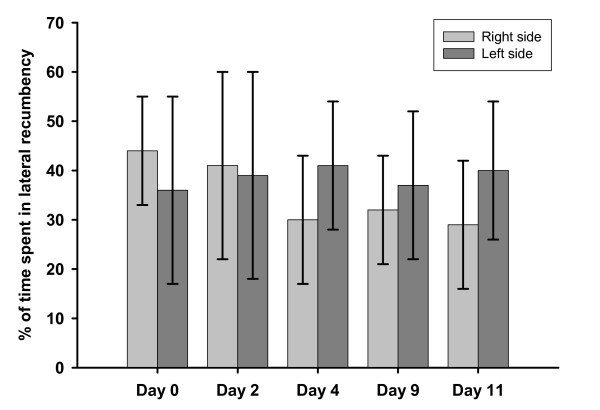
**Proportion of time spent in lateral recumbency on different days (T-day total), error bars indicating SD among sows**.

In general, total time in lateral recumbency was almost the same for the different days of observation, figure 2. However, the longest T-day total were observed on day 0 and day 2, when the sows spent on average 80% of the time in lateral recumbency (left and right side).

**Figure 3:**
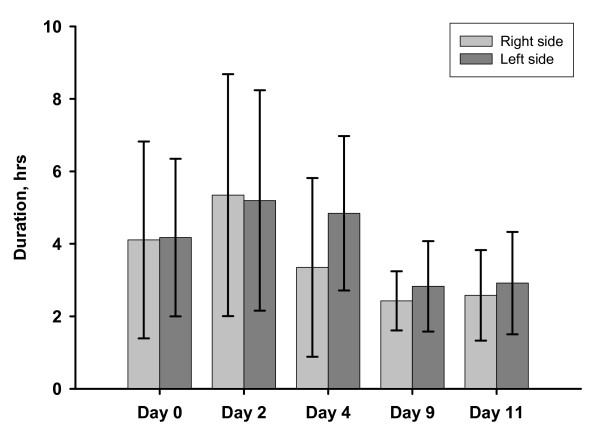
**Mean duration of the longest daily uninterrupted lying bout on different days (T-day max), error bars indicating SD among sows**.

Figure 3 shows the mean duration of T-day max for all sows. The longest daily bouts were observed on day 2, lasting for on average 5,3 hours (right side) and 5,2 hours (left side). Lying bout times decreased thereafter.

### Variation between the sows regarding T-max and T-total

**Figure 4:**
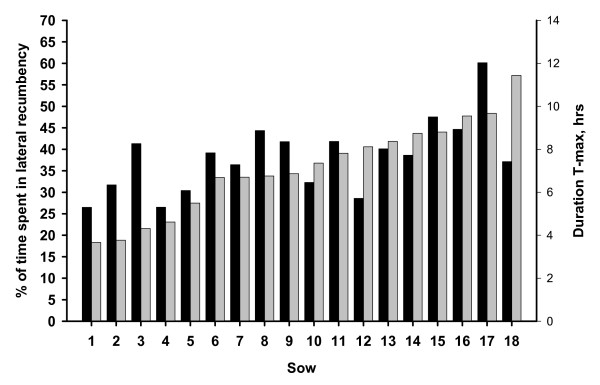
**Proportion of time spent in lateral recumbency (left side) during all days of observation (black) and the longest observed lying bout (T-max) recorded for left side (grey)**.

**Figure 5:**
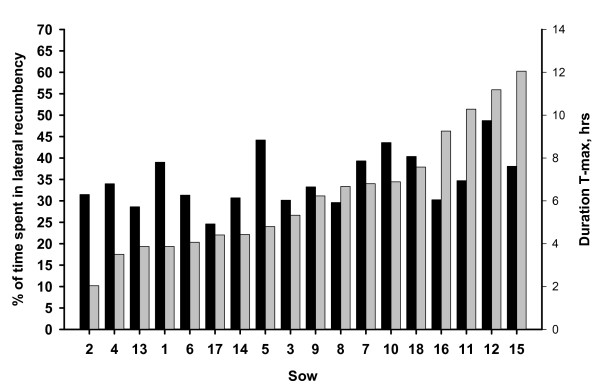
**Proportion of time spent in lateral recumbency (right side) during all days of observation (black) and the longest observed lying bout (T-max) recorded for right side (grey)**.

The mean duration of T-max for all sows were 7,2 hours (left side) respectively 6,3 hours (right side). Figure 4 and 5 shows the variation between sows regarding the duration of the longest observed lying bout and the proportion of time spent in lateral recumbency during all days of observation. Individual variation for T-max was slightly higher for the right side. In figure 4 it is shown that the sow with the longest T-max for left side, was lying for 11,4 hours (sow number 18). Sow number 17 had the largest proportion of time spent in lateral recumbency (60,1%). Regarding the right side, figure 5, the sow with the longest T-max was lying for 12,1 hours (sow number 15). Sow number 12 had the largest proportion of time spent in lateral recumbency (48,7%).

### Correlation between duration of longest lying bout and prevalence of shoulder lesions

**Figure 6:**
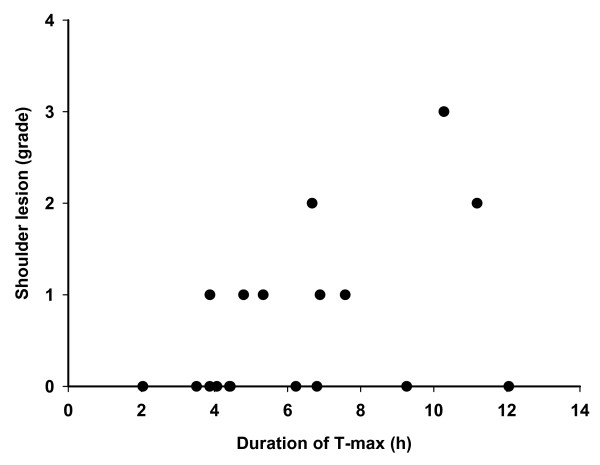
**Correlation between shoulder lesions (right side) and duration of the longest observed lying bout (T-max, right side)**.

A significant correlation was found between T-max for right side and the prevalence of shoulder lesions on the same side (Pearson's coefficient = 0.50; P = 0.049) (figure 6). This effect was only seen when the analysis was adjusted for back fat thickeness (mm) and straw supply (high vs. low) as partial factors. No significant effect was found for the left side.

When stratifying the data according to body condition (≤ 14 mm back fat vs. > 14 mm back fat) and straw supply at farrowing (low vs. high), it turned out that 7 out of 9 thin sows had aceess to the large amount of straw at farrowing. For sows in good condition it was the opposite. Only 3 of 9 sows were provided with the high straw supply at farrowing. Separate calculations for each stratum show a significant correlation between the longest observed lying bout (T-max, right side) and shoulder lesions recorded at the right side among sows in good condition with low supplies of straw (Spearman rank coefficient = 0,88; P = 0,021). The correlation was however not significant for the left side or among thin sows with high supplies of straw. Analysing the data from the other two strata ("thin sows with a low straw supply" and "sows in good conditioned with a high straw supply") was not meaningful since there were too few animals in those strata (n = 2 respectively 3 sows).

## Discussion

Several risk factors for development of shoulder lesions are reported in the literature. However, there is a lack of knowledge about how prolonged uninterrupted time spent in recumbency may affect the development. The results from this study support the hypothesis that the duration of uninterrupted lying bouts affects the risk for the development of shoulder lesions. This correlation could be confirmed between shoulder lesions at right shoulder and for the longest uninterrupted lying bout time (T-max). The mean duration of T-max for all sows were 7,2 hours (left side) respectively 6,3 hours (right side).

In several studies body condition has been pointed out as the most important risk factor for developing shoulder lesions [6,14]. One study showed that sows in body condition score lower than 3 at the time of weaning, had a higher risk to develop shoulder lesions than sows in body condition score 3 or more [13]. In our study, a significant correlation between the longest lying bout (T-max) and prevalence of shoulder lesions recorded on right side was observed for the sows in good body condition (backfat thickness > 14 mm at the time of entering the farrowing unit) and with low supplies of straw at farrowing. This correlation could however not be seen for the left side or among the sows in lower body condition (backfat thickness ≤ 14 mm) and with high supplies of straw. This indicates that even if thin sows are lying down only for short periods they are at risk due to the low body condition. Sows in good condition are partly protected by the layer of fat, but if too long uninterrupted periods are spent in lateral recumbency, the fat layer will not have a sufficient protective effect. Anatomical differences could be one reason why no significance correlation could be confirmed for left side. Which lying bout duration this critical point corresponds to is not investigated in this study. However, this critical point can be dependent on many factors such as anatomy, body condition and pressure. According to one study, lateral recumbency during 8 hours, with a pressure of 800 mm Hg resulted in injury on the skin [9].

Our findings in this limited data set suggest that it is the uninterrupted pressure on the tissue on the shoulder that may trigger shoulder lesions. To prevent shoulder lesions, the results of this study imply that it may be useful to activate the sow during the most inactive days, in order to diminish long lasting pressure on the shoulder. Pressure relief increases the blood flow in the tissue and thereby a lack of oxygen is avoided [12]. In a recent study of four herds during 18 months [19] it has been demonstrated that the occurrence of shoulder lesions was reduced when feeding the sows 5 to 8 times per day instead of 3. Hence, feeding regimes may be used to activate the sow and reduce the incidence of shoulder lesions.

In our study, only shoulder lesions which could be detected on the skin were classified. Even if no changes can be detected on the skin there can be injuries in the muscle tissue beneath the skin, which are not detectable without pathological inspection [9]. In the study eight of the eighteen sows developed detectable shoulder lesions (44%). This is a remarkable high value. However, the herd was chosen due to previous recorded high levels of shoulder lesions, which they now try to prevent. The sows in the herd consist of only purebred Landrace, which could be a related cause. Normally all the sows have a large amount of straw (approx. 15 kg) in the pen at the time of farrowing. Due to that another trial was running at the same time, some of the sows only got a small amount of straw which can have made them more vulnerable when lying on the concrete. The random distribution of straw supply (high vs. low) became uneven in relation to the body condition of the sows where most of the thin sows got a large amount of straw whereas the sows in good body condition got a small amount at the time of farrowing. Therefore no calculations to investigate the importance of straw were made. Many of the sows with a large amount of straw developed shoulder lesions but probably as a result of a low body condition score. This means that in this study, the presence of straw did not seem to prevent the development of shoulder lesion in thin sows, but this does not exclude that the straw can have a protective effect. It is possible that a larger amount of severe shoulder lesions (grade 3-4) may have been developed if the thin sows did not get access to the large amount of straw. In this study only two shoulder lesions of grade 3 were recorded. It was noticed that, in many pens where there was a large amount of straw, the straw was pushed away from the centre of the floor. This resulted in that the sows were lying on the concrete anyway. To get reliable answers about the effect of the straw, larger studies designed for studying this aspect are required.

## Conclusion

There is a relation between the duration of the longest uninterrupted lying bout time after farrowing and the occurrence of shoulder lesions in well conditioned sows provided with a small amount of straw present at time of farrowing. This suggests that avoiding prolonged uninterrupted recumbency would contribute to the prevention of the development of shoulder lesions in sows.

## Competing interests

The authors declare that they have no competing interests.

## Authors' contributions

ER contributed by gathering the data, pursuing the data analysis and writing the manuscript. RW participated in developing the design of the study, the gathering of data, the data analysis and the writing of the manuscript. BA suggested the hypothesis, contributed to the study design and the writing of the manuscript. All authors have read and approved the final manuscript.
